# Effect of Cooking Methods on Amphenicols and Metabolites Residues in Livestock and Poultry Meat Spiked Tissues

**DOI:** 10.3390/foods11213497

**Published:** 2022-11-03

**Authors:** Manli Wu, Xin Cheng, Xinyi Wu, Hang Qian, Wei Wang

**Affiliations:** 1 College of Food Science and Technology, Nanjing Agricultural University, Nanjing 210095, China; 2 National Center of Meat Quality and Safety Control, Nanjing Agricultural University, Nanjing 210095, China; 3 Key Laboratory of Animal Products Processing, Ministry of Agriculture and Rural Affairs, Nanjing 210095, China; 4 Jiangsu Collaborative Innovation Center of Meat Production and Processing, Quality and Safety Control, Nanjing Agricultural University, Nanjing 210095, China; 5 The Center for Agri-Food Quality & Safety, Ministry of Agriculture and Rural Affairs, Beijing 100125, China

**Keywords:** livestock and poultry meat, cooking methods, amphenicols, metabolites, residues

## Abstract

Foods of animal origin, as nutritional supplements, are usually consumed after cooking, but residues of amphenicols in fresh raw meat threaten human health. Therefore, this study was designed to evaluate the effects of boiling, deep-frying and microwave processing under different time conditions on the residue levels of amphenicols and metabolites in livestock and poultry meat. Antibiotic-free pork, beef, lamb and chicken samples were spiked with chloramphenicol (CAP), thiamphenicol (TAP), florfenicol (FF) and florfenicol amine (FFA) standard solutions and made into homogeneous meat blocks. These positive mock meat blocks were processed using three different cooking methods, and the analyses were performed by ultra-high-performance liquid chromatography-tandem mass spectrometry (UHPLC-MS/MS). The results showed that cooking methods, time and food matrices were the main factors influencing the changes in amphenicols and metabolites residues in livestock and poultry meat. With the increase in cooking time, boiling processing was the most effective in reducing the four drug residues in livestock and poultry meat matrices, followed by deep-frying, while microwaving caused an increase in drug residue concentrations. Although boiling and frying processes are effective strategies to reduce amphenicols and metabolites residues in meat, it cannot be assumed that these residues can always decrease to levels that are safe for consumer health, especially when the drug residue concentrations in raw meat are above the maximum residue limits (MRLs). Therefore, it is not reliable to remove residues of amphenicols and metabolites from food by cooking. The solution to the food safety problem of veterinary drug residues must start from the breeding source and accelerate the implementation of antibiotic reduction, antibiotic substitution and antibiotic-free farming.

## 1. Introduction

Livestock and poultry meat, rich in protein, fat, minerals and vitamins, is an important source of nutrients for the human body [1]. In recent years, livestock and poultry farming have grown rapidly worldwide. However, the industry is facing problems such as bacterial, viral and parasitic infections. If not given prompt treatment, they may lead to the occurrence of zoonotic diseases during the breeding process or through the food chain and cause severe economic losses. Thus, antibiotics are widely used in animal production to prevent and treat bacterial diseases, as well as to promote growth and improve feed utilization. They provide convenience to modern intensive farming and can meet the demand for livestock and poultry production and market consumption [2].

**Figure 1:**
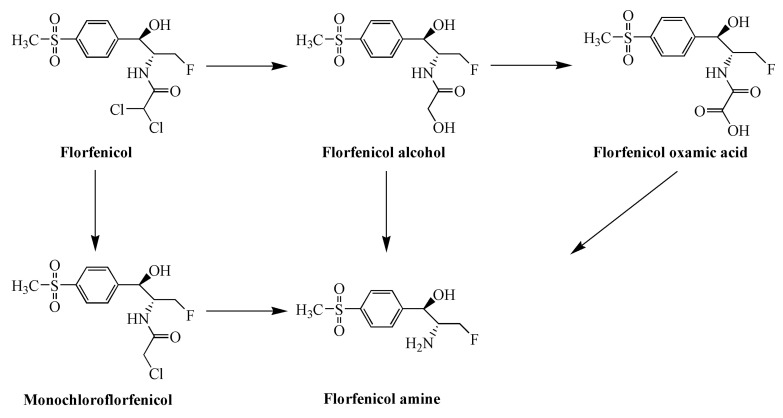
Florfenicol (FF) metabolic pathways.

Amphenicols, mainly including chloramphenicol (CAP), thiamphenicol (TAP) and florfenicol (FF), are a class of highly potent and economic antibiotics with broad-spectrum antimicrobial activities that are widely used in livestock and poultry breeding and production [3,4]. The first generation of amphenicols, CAP, has many toxic side effects and can disrupt the body’s hematopoietic function and trigger aplastic anemia (a condition in which the bone marrow fails to produce enough new cells to replenish the blood cells). It has been banned by China, the European Union, the United States and other countries and organizations for treating food-producing animals [5]. The para-nitro group on the aromatic ring of CAP is the leading group that causes aplastic anemia. As a derivative of CAP, TAP replaces the *p*-nitro (-NO_2_) of CAP with *p*-methylsulfonyl (-SO_2_CH_3_). The two have a similar antibacterial spectrum and antibacterial effect, but TAP is much less toxic than CAP. FF is a third-generation amphenicol drug obtained by replacing the C-3 hydroxyl group (-OH) of TAP with the fluorine atom. The presence of the fluorine atom reduces the number of sites for the acetylation of CAP and TAP by bacteria, thus enhancing the acetylation effect of the drug against bacterial resistance. FF has better antimicrobial activity, resistance and safety than TAP and CAP and is an animal-specific broad-spectrum antibiotic. The metabolic response of FF in the tested animals is shown in Figure 1, with the main metabolite being florfenicol amine (FFA) [6]. TAP and FF have completely replaced the use of CAP in food animals due to their excellent antibacterial effect and higher safety. Unfortunately, these antibiotics are often used irrationally, resulting in excessive residues in the tissues of the animals to be consumed, posing a significant challenge to food safety [7,8]. To protect consumers from potential health-related problems, many countries and organizations have established maximum residue limits (MRLs) for amphenicols in animal-origin foods (Table 1).

To date, food safety risk assessment, market supervision and the import/export certification of veterinary drug residues in livestock and poultry meat have been carried out on unprocessed products. However, most animal-derived foods are ordinarily cooked or processed prior to consumption to improve their nutrient digestibility, palatability and shelf-life [8]. Studies have shown that cooking not only affects nutrients such as protein and fat in livestock and poultry meat but also leads to changes in drug residue concentrations, chemical structures and their solubility in the tissues [9,10,11]. Therefore, to accurately assess the dietary exposure levels of drug residues, it is crucial to study the impacts of cooking methods on the residues of amphenicols and metabolites in livestock and poultry meat. Currently, there is minimal research on the changes in amphenicol antibiotic residues in livestock and poultry meat processing at home and abroad. The available studies also have problems, such as the single selection of meat species and cooking methods and unsystematic studies.

In this study, we added certain concentrations of CAP, TAP, FF and FFA standard solutions to the muscle of negative livestock and poultry (pig, cattle, sheep and chicken) and made meat blocks that were 18 g in size, had good drug homogeneity and were processed to simulate domestic cooking such as boiling, deep-frying and microwaving. Our aim was to assess the effects of three processing methods under different temporal conditions on the residue levels of amphenicols and metabolites in livestock and poultry meat. The development of this study may provide some data basis and theoretical support for the accurate assessments of meat safety and the risk of dietary exposure to amphenicol antibiotics.

## 2. Materials and Methods

### 2.1. Samples

Fresh raw livestock and poultry meat (pork, beef, lamb and chicken) were provided by the Supervision, Inspection and Testing Center for Quality of Meat-Products, Ministry of Agriculture and Rural Affairs (Nanjing, China). All samples tested negative for residues of amphenicols and metabolites before the experiment.

### 2.2. Chemicals and Reagents

Standards of CAP, TAP, FF, FFA and CAP-D5 (purity ≥99.50%) were obtained from Dr. Ehrenstorfer GmbH (Augsburg, Germany). FFA-D3 standard (purity ≥99.50%) was purchased from Toronto Research Chemicals (North York, ON, Canada). HPLC-grade solvents, including methanol, acetonitrile (ACN), ethyl acetate and n-hexane, were supplied by Merck Company (Darmstadt, Germany). HPLC-grade acetone, formic acid and guaranteed reagent grade ammonium hydroxide were provided by Sinopharm Chemical Reagent Co., Ltd. (Shanghai, China). Ultrapure water was supplied by the Sartorius-Arium pro system (Sartorius AG, Goettingen, Germany).

Individual standard stock solutions (1 mg/mL) of CAP, TAP, FF, FFA, CAP-D5 and FFA-D3 were prepared in methanol and stored at −20 °C for six months. CAP-D5 and FFA-D3 were used as internal standards. The combined standard working solution at a concentration of 10 μg/mL for each analyte was prepared by mixing equal volumes of the individual standard stock solution of CAP, TAP, FF and FFA with a concentration of 1 mg/mL and diluting with methanol. The mixed internal standard working solution at the concentration of 1.0 μg/mL was prepared by diluting the internal standard stock solutions (1 mg/mL) with methanol. The abovementioned standard working solutions were stored at −20 °C for up to three months.

### 2.3. Preparation of Positive Mock Samples

Fresh negative pork, beef, lamb and chicken samples with fat and connective tissue removed were weighed and then homogenized by the addition of standards of amphenicols and metabolites, respectively. Where CAP was spiked at 20 μg/kg in the four kinds of livestock and poultry meat, TAP, FF and FFA were spiked at twice the MRLs specified in the Chinese standard GB 31650-2019 “National food safety standard—Maximum residue limits for veterinary drugs in foods” (Table 2) [12]. Next, the spiked and homogenized meat mash was prepared into cuboid meat blocks with a length × width × height of about 25 mm × 25 mm × 33 mm and a mass of about 18 g for subsequent cooking.

### 2.4. Cooking Operations

According to the cooking method of this experiment, the positive simulated livestock and poultry meat blocks of each matrix (pork, beef, lamb and chicken) were divided into three groups of 21 portions each. Before further treatment, three pieces of meat blocks were randomly selected from each group to verify the spiking homogeneity of the positive mock samples. The remaining pieces were used for boiling, deep-frying and microwave processing, respectively.

#### 2.4.1. Boiling

The boiling process was performed at 100 °C in a water bath (TW20, Julabo Laborthechnik GmbH, Seelbach, Germany) for 5, 10, 15, 20 and 25 min, respectively, and then it was allowed to cool naturally to room temperature (22 ± 2 °C) before being weighed and subsequently detected and analyzed within one day. Three parallel experiments were conducted at each time point, with the unprocessed meat blocks serving as the control group.

#### 2.4.2. Deep-Frying

The meat pieces were fried with edible oil at 180 °C, turned over every 30 s, taken out at 1, 2, 3, 4 and 5 min, respectively, and allowed to cool naturally to room temperature (22 ± 2 °C) before being weighed and subsequently detected and analyzed within one day. Three parallel experiments were conducted at each time point, with the unprocessed meat blocks serving as the control group.

#### 2.4.3. Microwaving

The microwaving operation was carried out in a turntable domestic microwave oven (P70D20TL-D4, Guangdong Galanz Microwave Electrical Appliances Manufacturing Co., Ltd., Guangdong, China). The meat pieces were cooked under full power (700 W, 2450 MHz) for 0.25, 0.50, 0.75, 1.00 and 1.25 min, respectively, and allowed to cool naturally to room temperature (22 ± 2 °C) before being weighed and subsequently detected and analyzed within one day. Three parallel experiments were conducted at each time point, with the unprocessed meat blocks serving as the control group.

The changes in the residual concentrations of amphenicols and metabolites in the livestock and poultry meat blocks after cooking were calculated as follows:

(1) $$\Delta T\;\left(\%\right)=\left|\frac{C_{P}-C_{0}}{C_{0}}\right|\times 100$$

where *C*_0_ (μg/kg) is the initial concentration of drug residues in the uncooked livestock and poultry pieces; *C_p_* (μg/kg) is the concentration of drug residues in the cooked livestock and poultry pieces.

### 2.5. Sample Preparation and Analysis

#### 2.5.1. Sample Preparation

The extraction and purification of amphenicols and metabolites from livestock and poultry meat were performed using our self-built method [13]. Briefly, pork, beef, lamb and chicken samples were chopped and homogenized at 10,000 r/min using an HM6300 intelligent homogenizer (Lab Precision Beijing Technology Co., Ltd., Beijing, China). After homogenization, 5 g (accuracy, 0.01 g) of each livestock and poultry meat sample were placed in a 50 mL centrifuge tube. Then, 10 μL of 1.0 μg/mL mixed internal working standard solution and 15 mL of ethyl acetate with 2% ammonia were added and vortexed for 1 min. The mixture was centrifuged at 4 °C for 5 min at 10,621 g in a refrigerated centrifuge (D-16C, Sartorius Lab Instruments GmbH & Co. KG, Goettingen, Germany), and the supernatant was collected. The extraction operation was repeated with another 15 mL of ethyl acetate with 2% ammonia, and the supernatants were combined. The supernatants were evaporated to dryness at 40 °C with a nitrogen evaporator (N-EVAPTM-112, Organomation Associates Inc., Berlin, MA, USA). Subsequently, the residue was reconstituted with 5 mL of acetone: n-hexane (1:9, *v/v*), purified by a CNW Si solid-phase extraction (SPE) column and defatted with ACN-saturated hexane. Finally, the solution was filtered through a 0.22 μm filter membrane and injected into the ultra-high-performance liquid chromatography-tandem mass spectrometry (UHPLC-MS/MS) system analysis.

#### 2.5.2. UHPLC-MS/MS Conditions

The separation and quantification of the four amphenicols and metabolites were performed on a Thermo Scientific Vanquish ultra-high-performance liquid chromatography instrument coupled with a Thermo Scientific TSQ Quantis mass spectrometer (Thermo Fisher Scientific, Waltham, MA, USA). A Waters Acquity UPLC HSS C18 (2.1 mm × 50 mm, 1.8 μm) was used as the analytical column, with a Waters Acquity UPLC HSS C18 VanGuard precolumn (2.1 mm × 5 mm, 1.8 μm) attached to the front end. The column temperature was 40 °C, and the injection volume was 2.0 μL. Gradient elution, with water and ACN as mobile phases, was carried out at a constant flow rate of 0.3 mL/min. The starting mobile phase composition was 4:96 (ACN/water) at 0 min. It was switched to 96:4 after 4 min and held for 2 min, returning to the initial conditions at 10 min.

The MS/MS was equipped with an ESI source and scanned in positive ion (PI) and negative ion (NI) mode switching. CAP, TAP, FF and CAP-D5 were analyzed in NI mode, while FFA and FFA-D3 were analyzed in PI mode. The detection mode was selective reaction monitoring. The ESI source was operated with the following capillary voltages: 3.5 kV in PI mode, 2.5 kV in NI mode; sheath gas: 50 Arb; auxiliary gas: 10 Arb; ion transfer tube temperature: 325 °C; and evaporator temperature: 350 °C. The specific mass spectrometry parameters of amphenicols and metabolites are shown in Table 3. Under these conditions, the limits of detection and limits of quantification for all four analytes were below 1.5 µg/kg and 5.0 µg/kg, respectively, with good precision (RSD < 9.0%) and accuracy (recovery > 72.0%). This means that the extraction and purification procedures used in this experiment are effective and that the UHPLC-MS/MS conditions are applicable to detecting residues of amphenicols and metabolites in livestock and poultry meat samples. The linear working ranges of the current quantitative method were 5.0–50.0 μg/kg for CAP, 1–100 μg/kg for TAP and 5–300 μg/kg for FF and FFA, at which concentration ranges the changes in the peak area were proportional to the changes in the drug concentrations (R^2^ > 0.9990 for CAP, TAP, FF and FFA).

### 2.6. Statistical Analysis

Data acquisition was performed using TraceFinder software (version number: 4.1.31.9, Thermo Fisher Scientific, Waltham, MA, USA). Three parallels were carried out for each experiment, and the data were expressed as the mean ± standard deviation. Statistical analysis was performed using SAS software (version number: 6.2.9200, SAS Institute Inc., Cary, NC, USA), and the significant influence of different cooking methods or times on the concentrations of amphenicols and metabolites in livestock and poultry meat blocks were analyzed by one-way analysis of variance (ANOVA) and Duncan’s multiple comparisons. A *p* < 0.05 was considered to be statistically significant. The figures were plotted using OriginPro 2022 software (OriginLab, Northampton, MA, USA).

## 3. Results and Discussion

### 3.1. Homogeneity Analysis of Positive Simulated Samples

Studies related to the effect of thermal processing on drug residues have shown significant differences in the percentage of thermal degradation of amphenicols in model solutions (water), spiked tissues and incurred samples, and their degradation products vary. To provide reliable information on the stability of residues of amphenicols for food safety risk assessments, Tian [14] suggested that incurred samples should be systematically implemented rather than spiked tissues to study the impact of cooking on drug residues. However, the subject of this experiment was livestock and poultry meat, and positive samples contaminated with amphenicols and metabolites from the market were difficult to collect. Furthermore, livestock and poultry animals bioaccumulate slowly, and it is also challenging to obtain contamination through controlled laboratory conditions. Therefore, in this study, positive mock samples could only be obtained by adding amphenicols and metabolites standards to negative livestock and poultry meat samples.

In order to ensure the consistency of the target compound concentrations in the meat blocks used for subsequent cooking, the homogeneity analysis of positive mock samples was carried out in this experiment, and the results are shown in Table 4. The one-way ANOVA showed *p* > 0.05 for the measured concentrations of amphenicols and metabolites in pork, beef, lamb and chicken blocks, indicating that the differences in drug concentrations between meat nuggets were insignificant. That is, the meat nuggets prepared by this experimental method had a good homogeneity and could meet the requirements for subsequent cooking.

### 3.2. Processing Quality Loss of Livestock and Poultry Meat

**Figure 2:**
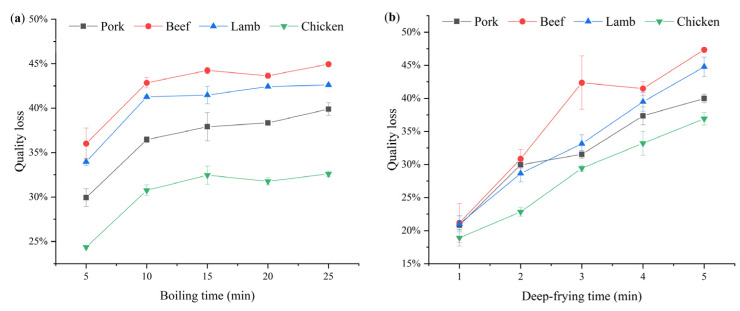

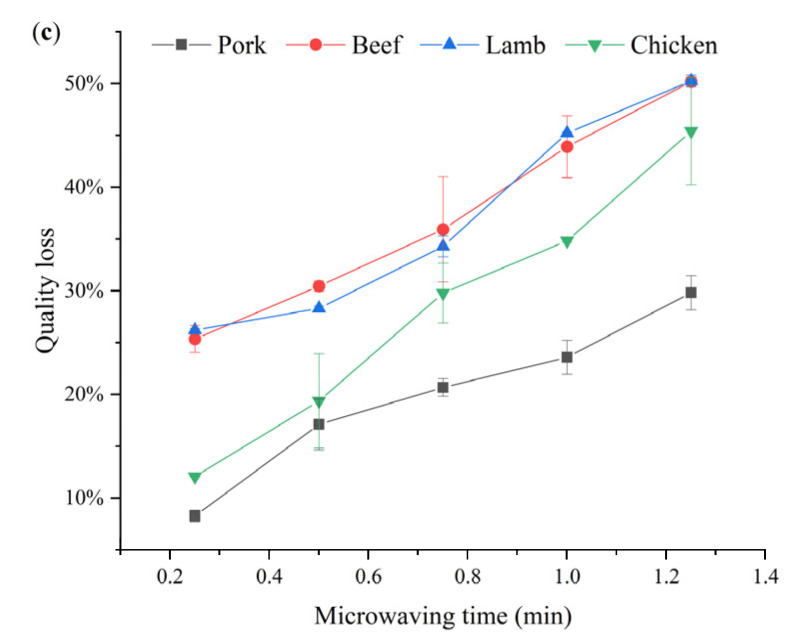
Processing quality loss of livestock and poultry meat. (**a**) Boiling; (**b**) Deep-frying; (**c**) Microwaving.

The effects of boiling, deep-frying and microwave processing on the quality loss of livestock and poultry meat are shown in Figure 2. The mass loss of livestock and poultry meat during boiling showed an overall trend of rising and then leveling off with time. After 20 min of boiling, the quality of pork, beef, lamb and chicken remained stable (*p* > 0.05). During deep-frying and microwave processing, livestock and poultry meat quality loss continued to increase over time (*p* < 0.05). The quality losses of pork, beef, lamb and chicken were 39.89%, 44.95%, 42.61% and 32.60% and 39.98%, 47.34%, 44.76% and 36.91% at 25 min of boiling and 5 min of deep-frying, respectively, and the degree of loss was similar for both. At 1.25 min of microwaving, the quality loss of the four livestock and poultry meat species reached 29.82%, 50.19%, 50.26% and 45.41%, respectively. Compared with boiling and deep-frying, the quality loss rate was faster in microwaves. The reason is that microwaves can heat the whole material simultaneously, which results in a violent heating process, rapid temperature rise and faster water evaporation, thus causing the most severe quality loss in a short time. The determination of the quality loss index should facilitate the understanding of the effect of subsequent cooking on the concentration of drug residues in livestock and poultry meat.

### 3.3. Effect of Cooking Time on Residues of Amphenicols and Metabolites in Livestock and Poultry Meat

The residue levels of amphenicols and metabolites in the meat blocks of livestock and poultry cooked by boiling for varying time periods are presented in Table 5. It can be seen from the table that the residue concentrations of CAP, TAP, FF and FFA in pork, beef, lamb and chicken gradually decreased with the prolonged boiling time. Within 25 min, the depletion rates of the four drugs were 38.55–75.75% in pork, 47.60–100% in beef, 20.18–100% in lamb and 39.31–50.19% in chicken. This is consistent with the results reported by Shakila et al. [15] and Filazi et al. [10], which showed that boiling reduced CAP residues in shrimps and FF and FFA residues in eggs, and the loss was strongly correlated with heating time. In addition, Table 5 also shows that the elimination rates of the four drugs in different livestock and poultry meat matrices were different during the boiling process. CAP, TAP and FF were removed faster in beef and lamb and relatively slower in pork and chicken, while FFA was removed faster in beef, pork and chicken and was removed the slowest in lamb.

The effects of different deep-frying times on the residue levels of the four amphenicols and metabolites in livestock and poultry meat are shown in Table 6. The residue concentrations of CAP and TAP in pork, beef, lamb and chicken showed a decreasing or first increasing and then decreasing trend with frying time, while FF and FFA showed a decreasing trend in all four livestock and poultry meats. These findings indicate that the effect of heat treatment on amphenicols and metabolites is matrix-dependent. In addition, the reason for the elevated CAP and TAP residue concentrations in some livestock and poultry meat at the beginning of the deep-frying process may be related to the rapid water loss and evaporation from the meat at the initial stage with less drug loss [16]. Residue concentrations of CAP, TAP, FF and FFA in pork decreased by 6.70–41.29% within 5 min of frying, while those in beef, lamb and chicken decreased by 43.07–61.14%, 5.45–57.16% and 15.20–40.27%, respectively. One notable result worth emphasizing was that all four drugs were removed at the fastest rate in beef during deep-frying, as in the case of boiling. The reason may be that beef has a high water content, and heating results in the most severe loss of quality (Figure 2) due to the disruption of its water-retaining protein spatial structure, the tightening of myogenic fibers and reduced water-binding capacity [17,18]. It is known that the decrease in the water binding capacity of meat increases drug degradation [19]. Therefore, it can be speculated that the highly reduced contents of amphenicols and metabolites in beef may be at least partially attributed to the significant decrease in the water-binding capacity of the meat caused by heating. In addition, the thermal treatment itself can affect the drug’s chemical structure and its solubility in tissues [9].

It is evident from the above that boiling and deep-frying can effectively reduce the concentrations of four amphenicols and metabolites in livestock and poultry meat and that drug residue levels continue to decrease with the prolongation of cooking time. The loss of amphenicols and metabolites during cooking questions their stability when heated. Shakila et al. [15] have reported that CAP is an unstable drug destroyed during cooking and boiling. Tian [20] detected seven degradation products and metabolites of CAP in cooked mussels containing CAP, and structures were proposed for six. Similarly, Franje et al. [11] demonstrated that FF residues in chicken meat degraded to produce TAP in water at 100 °C by identifying the degradation structures of amphenicols after processing. The loss of amphenicols and metabolites observed in the present study after boiling and deep-frying suggests that they might have been destroyed or degraded to other substances. Moreover, it is also possible that the drug migrated from the livestock and poultry meat tissue into the surrounding liquid or meat juices during cooking, resulting in decreased residual concentration.

There was a difference in the effect of microwaving on the concentration of residues of amphenicols and metabolites in livestock and poultry meat over time compared with boiling and deep-frying (Table 7). From 0 to 1.25 min, CAP, TAP, FF and FFA showed an increasing or first decreasing and then increasing trend in pork, beef, lamb and chicken. At 1.25 min, the concentrations of all four drugs increased in the livestock and poultry meat matrices compared with the control groups. The increase rate was 8.43–43.84% for pork, 23.16–33.27% for beef, 6.50–80.29% for lamb and 26.66–135.92% for chicken. This result is inconsistent with the previous proposal by Nashwa et al. [21] that microwave heating was the most effective method for reducing drug residues in meat. In this experiment, microwaves did not cause a reduction in the levels of residues of amphenicols and metabolites. The reason for this may be that microwave processing caused a large amount of rapid water evaporation from livestock and poultry meat and a serious loss of quality (Figure 2c), which significantly reduced the water content of the meat while the drug abatement was at a low level. Overall, the livestock and poultry meat matrices are equivalent to being concentrated, and, therefore, the concentration of drug residues in the samples was elevated [22].

### 3.4. Effect of Cooking Methods on Residues of Amphenicols and Metabolites in Livestock and Poultry Meat

**Figure 3:**
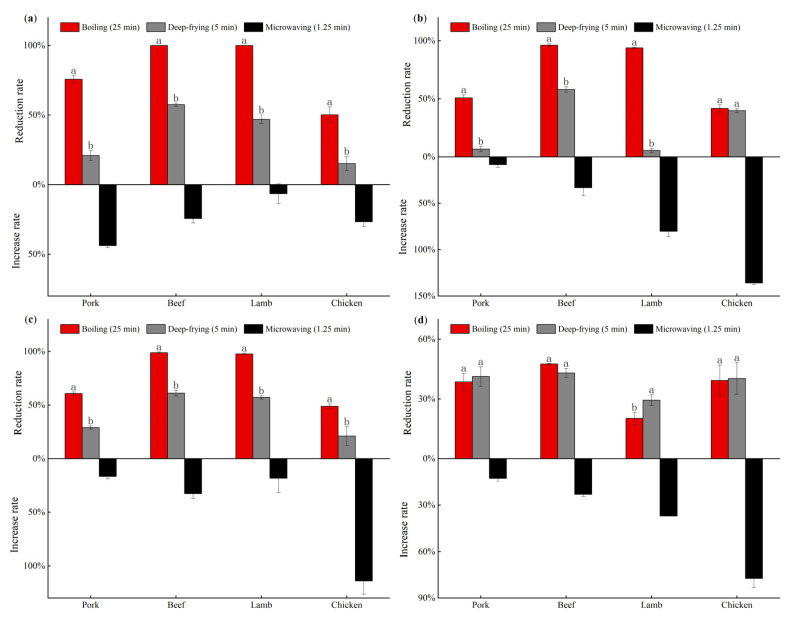
The rate of change in residue concentrations of amphenicols and metabolites in livestock and poultry meat under different cooking methods. (**a**) CAP; (**b**) TAP; (**c**) FF; (**d**) FFA. Different lowercase letters represent significant differences (*p* < 0.05) in the reduction rate of the same drug in the same species of livestock and poultry meat under boiling (25 min) and deep-frying (5 min) treatments. The same lowercase letter indicates no significant difference (*p* > 0.05).

In order to compare the effects of different cooking methods on the concentration changes of amphenicols and metabolites in livestock and poultry meat, each cooking endpoint was selected for analysis in this study (Figure 3). Among the three cooking methods, microwaving increased the concentration of the four drug residues in the meat matrices of livestock and poultry, while boiling and deep-frying had the opposite effect, and the removal effects of the two were also different. Figure 3 illustrates that the removal rates of CAP and FF in four types of livestock and poultry meat and of TAP in pork, beef and lamb by boiling were significantly higher than those in deep-frying (*p* < 0.05), but there was no significant difference between the two for TAP in chicken (*p* > 0.05). In terms of FFA, there was no significant difference between boiling and deep-frying in pork, beef and chicken (*p* > 0.05), while the removal rate of boiling was lower than that of deep-frying in lamb (*p* < 0.05). These results have shown that different cooking methods have different effects on the removal of amphenicols and metabolites from livestock and poultry meat. Boiling showed the highest reduction effect on the drug residues in livestock and poultry meat matrices, followed by deep-frying, while microwaving caused an increase in drug residue concentrations. Based on the previous reports, we speculate that the reduction in drug residue concentrations in the matrices by boiling and deep-frying may be related to moisture loss, drug migration and degradation [11,23,24]. In addition, the overall removal rate of amphenicols and metabolites in livestock and poultry meat observed in this experiment was higher with boiling than with frying. The reason for this may be that, on the one hand, the heating rate of deep-frying is faster than that of boiling, less water is lost in the form of transfer in deep-frying than in boiling for the same degree of mass loss (Figure 2) and less of the drug is lost with it. On the other hand, deep-frying may create a hard crust on the surface of the meat, which, in turn, slows down the rate of drug loss with moisture [25].

## 4. Conclusions

According to the Procedural Manual of the Codex Alimentarius Commission (2018), dietary exposure assessments of contaminants in livestock and poultry meat should consider the presence of these contaminants in raw meat as a potential source and account for the effect of food cooking and processing on drug residues. The results of this experiment indicated that the changes in drug residues in livestock and poultry meat depended on the cooking time, the methods and the type of food matrices. Under both boiling and deep-frying cooking methods, extended heating time effectively reduced the concentration of the four drug residues, thereby reducing the risk of dietary exposure to consumers. However, microwaving led to increased drug residue concentrations. Although boiling and deep-frying cooking are both effective ways to reduce residues of amphenicols and metabolites in meat, there is no guarantee that these residues will always decrease to a safe level in terms of consumer health, especially when drug residue concentrations in raw livestock and poultry meat are higher than the MRLs. In summary, from the safety and toxicological point of view, it is unsafe to rely on cooking to remove residues of amphenicols and metabolites from food. The solution to the food safety problem of veterinary drug residues must start at the source—from the production, operation and use of veterinary drugs—to strengthen supervision in order to establish a regulated veterinary drug market order.

## Figures and Tables

**Table 1:** Maximum residue limits (MRLs) for amphenicols and metabolites in animal-derived foods.

Compound	Species	Target Tissue	MRL (µg/kg)		
			China	U.S.	EU
CAP	All species	All tissues			
TAP	All species	All tissues	50		50
FF (sum of FF and FFA)	Porcine	Skin and fat	500		500
		Muscle	300	200	300
		Liver	2000	2500	2000
		Kidney	500		500
	Bovine	Muscle	200	300	200
		Liver	3000	3700	3000
		Kidney	300		300
	Ovine caprine	Muscle	200		200
		Liver	3000		3000
		Kidney	300		300
	Poultry	Skin and fat	200		200
		Muscle	100		100
		Liver	2500		2500
		Kidney	750		750

CAP: chloramphenicol; TAP: thiamphenicol; FFA: florfenicol amine; U.S.: the United States; EU: the European Union.

**Table 2:** Spiked concentrations of amphenicols and metabolites in positive mock samples.

Matrix	Concentration (μg/kg)			
	CAP	TAP	FF	FFA
Pork	20	100	300	300
Beef	20	100	200	200
Lamb	20	100	200	200
Chicken	20	100	100	100

**Table 3:** Mass spectrometry parameters of amphenicols and metabolites.

Compound	Reaction Mode	Precursor Ion (*m*/*z*)	Product Ion (*m*/*z*)	Collision Voltage (V)	Radio-Frequency Voltage (V)
CAP	−	321	257	16.94	146
			152 *	10.23	146
CAP-D5	−	326	157	16.94	146
TAP	−	354	290 *	20.08	164
			185	12.39	164
FF	−	356	185 *	31.87	141
			119	19.17	141
FFA	+	248	230 *	24.52	97
			130	12.88	97
FFA-D3	+	251	233	12.88	97

*: quantitative ion; −: negative ion reaction mode; +: positive ion reaction mode.

**Table 4:** Homogeneity analysis of amphenicols and metabolites added to livestock and poultry meat blocks.

Matrix	Compound	Group	Concentration (μg/kg)				*p*-Value
			No. 1	No. 2	No. 3	Average	
Pork	CAP	P-1	20.12	18.18	19.60	19.58	0.89
		P-2	20.72	19.73	18.51		
		P-3	18.05	19.99	21.35		
	TAP	P-1	107.28	113.02	94.49	102.30	0.73
		P-2	91.91	91.25	112.76		
		P-3	110.56	95.66	103.77		
	FF	P-1	308.13	302.82	292.61	293.95	0.18
		P-2	288.18	301.16	292.05		
		P-3	296.31	277.32	286.97		
	FFA	P-1	314.81	312.97	310.16	303.21	0.09
		P-2	287.32	295.51	306.97		
		P-3	294.91	296.31	309.97		
Beef	CAP	B-1	19.89	20.12	19.97	19.75	0.85
		B-2	18.46	19.43	21.33		
		B-3	18.33	19.90	20.30		
	TAP	B-1	99.38	93.05	99.50	97.44	0.84
		B-2	91.91	101.25	102.21		
		B-3	95.61	96.60	97.46		
	FF	B-1	190.30	200.80	199.90	199.53	0.31
		B-2	198.22	205.55	205.34		
		B-3	196.01	197.65	201.98		
	FFA	B-1	194.87	210.69	201.79	200.43	0.71
		B-2	197.92	192.11	204.15		
		B-3	197.13	198.90	206.31		
Lamb	CAP	L-1	18.31	18.47	20.34	19.66	0.45
		L-2	18.50	19.88	20.95		
		L-3	20.01	19.62	20.86		
	TAP	L-1	103.25	101.34	96.85	99.44	0.76
		L-2	97.44	98.22	101.75		
		L-3	96.21	97.60	102.31		
	FF	L-1	196.56	200.60	199.40	199.61	0.90
		L-2	189.97	204.78	203.11		
		L-3	202.67	198.46	200.90		
	FFA	L-1	197.48	198.90	201.50	200.52	0.57
		L-2	201.19	196.40	204.10		
		L-3	203.46	200.79	200.90		
Chicken	CAP	C-1	22.30	20.10	20.60	20.59	0.58
		C-2	20.60	19.87	20.70		
		C-3	19.90	21.10	20.10		
	TAP	C-1	100.50	99.90	101.70	100.41	0.36
		C-2	104.20	103.60	97.86		
		C-3	98.66	95.98	101.30		
	FF	C-1	99.50	102.88	99.65	100.20	0.07
		C-2	101.60	103.00	100.90		
		C-3	97.40	96.80	100.10		
	FFA	C-1	104.60	97.00	100.90	101.31	0.87
		C-2	103.92	98.00	104.30		
		C-3	101.30	99.70	102.10		

Positive simulated meat blocks of each matrix (pork, beef, lamb and chicken) were divided into three groups corresponding to the subsequent boiling, deep-frying and microwaving treatment groups. The three groups for pork were numbered P-1, P-2 and P-3, respectively. The three groups for beef were numbered B-1, B-2 and B-3, respectively. The three groups for lamb were numbered L-1, L-2 and L-3. The three groups for chicken were numbered C-1, C-2 and C-3. No. 1, No. 2 and No. 3 were the three meat blocks randomly selected from each group.

**Table 5:** Effect of boiling time on residues of amphenicols and metabolites in livestock and poultry meat.

Compound	Time (min)	Concentration (μg/kg) [Reduction Rate (%)]			
		Pork	Beef	Lamb	Chicken
CAP	0	19.30 ± 1.00 ^a^	19.99 ± 0.12 ^a^	19.04 ± 1.13 ^a^	21.00 ± 1.15 ^a^
	5	20.37 ± 0.86 ^a^ (−5.54)	14.64 ± 1.62 ^b^ (26.76)	19.33 ± 0.63 ^a^ (−1.52)	19.80 ± 1.80 ^a^ (5.71)
	10	13.04 ± 0.73 ^b^ (32.44)	3.43 ± 0.34 ^c^ (82.84)	6.29 ± 0.76 ^b^ (66.96)	16.82 ± 0.70 ^b^ (19.90)
	15	10.24 ± 1.05 ^c^ (46.94)	0.92 ± 0.65 ^d^ (95.40)	2.01 ± 0.34 ^c^ (89.44)	15.57 ± 0.11 ^b^ (25.86)
	20	7.36 ± 0.46 ^d^ (61.87)	0.00 ± 0.00 ^e^ (100.00)	0.32 ± 0.12 ^d^ (98.32)	11.30 ± 0.32 ^c^ (46.19)
	25	4.68 ± 0.54 ^e^ (75.75) *	0.00 ± 0.00 ^e^ (100.00)	0.00 ± 0.00 ^d^ (100.00)	10.46 ± 1.13 ^c^ (50.19) *
TAP	0	104.93 ± 9.49 ^a^	97.31 ± 3.69 ^a^	100.48 ± 3.29 ^a^	100.70 ± 0.92 ^a^
	5	88.85 ± 2.92 ^b^ (15.32)	76.44 ± 5.53 ^b^ (21.45)	67.28 ± 0.34 ^b^ (33.04)	83.50 ± 8.66 ^b^ (17.08)
	10	77.53 ± 2.22 ^c^ (26.11)	32.43 ± 0.33 ^c^ (66.67)	39.75 ± 3.85 ^c^ (60.44)	73.84 ± 1.96 ^c^ (26.67)
	15	69.96 ± 1.35 ^d^ (33.33)	16.29 ± 1.31 ^d^ (83.26)	23.17 ± 0.24 ^d^ (76.94)	70.80 ± 4.16 ^c^ (29.69)
	20	55.00 ± 1.37 ^e^ (47.58)	7.13 ± 2.58 ^e^ (92.67)	11.77 ± 0.70 ^e^ (88.29)	56.22 ± 0.91 ^d^ (44.17)
	25	51.59 ± 2.60 ^e^ (50.83) *	3.70 ± 1.12 ^e^ (96.20)	6.11 ± 0.64 ^f^ (93.92)	58.70 ± 3.07 ^d^ (41.71) *
FF	0	301.19 ± 7.89 ^a^	197.00 ± 5.82 ^a^	198.85 ± 2.07 ^a^	100.68 ± 1.91 ^a^
	5	286.97 ± 7.54 ^a^ (4.72)	114.19 ± 4.97 ^b^ (42.04)	185.27 ± 8.69 ^b^ (6.83)	82.63 ± 4.71 ^b^ (17.93)
	10	220.32 ± 0.51 ^b^ (26.85)	37.53 ± 2.26 ^c^ (80.95)	83.78 ± 5.34 ^c^ (57.87)	71.80 ± 0.65 ^c^ (28.68)
	15	188.13 ± 9.59 ^c^ (37.54)	12.70 ± 3.59 ^d^ (93.55)	38.13 ± 3.10 ^d^ (80.82)	65.95 ± 2.67 ^d^ (34.50)
	20	142.46 ± 1.93 ^d^ (52.70)	3.70 ± 1.91 ^e^ (98.12)	12.22 ± 0.09 ^e^ (93.85)	51.73 ± 1.97 ^e^ (48.62)
	25	118.41 ± 4.60 ^e^ (60.69) *	2.30 ± 1.62 ^e^ (98.83)	4.56 ± 1.45 ^e^ (97.71)	51.52 ± 2.23 ^e^ (48.83) *
FFA	0	312.65 ± 2.34 ^a^	202.45 ± 7.93 ^a^	199.29 ± 2.04 ^a^	100.83 ± 3.80 ^a^
	5	265.97 ± 14.91 ^b^ (14.93)	130.20 ± 10.82 ^b^ (35.69)	207.13 ± 8.47 ^a^ (−3.93)	100.62 ± 14.12 ^a^ (0.21)
	10	244.29 ± 2.32 ^c^ (21.86)	117.30 ± 18.61 ^bc^ (42.06)	197.65 ± 4.55 ^a^ (0.82)	98.52 ± 7.92 ^a^ (2.29)
	15	212.98 ± 2.88 ^d^ (31.88)	111.86 ± 11.21 ^bc^ (44.75)	181.69 ± 7.05 ^ab^ (8.83)	84.83 ± 3.05 ^ab^ (15.87)
	20	215.14 ± 10.48 ^d^ (31.19)	106.62 ± 5.62 ^c^ (47.34)	150.78 ± 7.46 ^b^ (24.34)	80.22 ± 10.65 ^ab^ (20.44)
	25	192.12 ± 13.04 ^e^ (38.55) *	106.09 ± 19.02 ^c^ (47.60)	159.07 ± 6.05 ^b^ (20.18)	61.19 ± 2.12 ^b^ (39.31) *

Different lowercase letters in the same column indicate significant differences (*p* < 0.05) in the concentration of the same compound in the same species of livestock and poultry meat between different boiling times. * indicates that the drug residue concentrations do not meet the requirements of non-detectable (CAP) or lower than MRLs (TAP, FF (sum of FF and FFA)), as stipulated by the Chinese standard GB 31650-2019 and (EU) No 37/2010 after boiling.

**Table 6:** Effect of deep-frying time on residues of amphenicols and metabolites in livestock and poultry meat.

Compound	Time (min)	Concentration (μg/kg) [Reduction Rate (%)]			
		Pork	Beef	Lamb	Chicken
CAP	0	19.65 ± 1.11 ^bc^	19.74 ± 1.46 ^a^	19.78 ± 1.23 ^b^	20.39 ± 0.45 ^a^
	1	25.18 ± 0.25 ^a^ (−28.14)	19.83 ± 1.18 ^a^ (−0.46)	22.38 ± 0.68 ^a^ (−13.14)	20.79 ± 0.41 ^a^ (−1.96)
	2	20.71 ± 0.51 ^b^ (−5.39)	13.81 ± 0.19 ^b^ (30.04)	17.72 ± 0.41 ^c^ (10.41)	19.09 ± 0.79 ^ab^ (6.38)
	3	19.74 ± 1.18 ^bc^ (−0.46)	14.16 ± 0.22 ^b^ (28.27)	14.51 ± 0.09 ^d^ (26.64)	18.94 ± 1.03 ^ab^ (7.11)
	4	18.81 ± 0.87 ^c^ (4.27)	9.57 ± 0.77 ^c^ (51.52)	12.52 ± 0.94 ^e^ (36.70)	18.36 ± 1.17 ^b^ (9.96)
	5	15.53 ± 0.71 ^d^ (20.97) *	8.40 ± 0.23 ^c^ (57.45) *	10.50 ± 0.61 ^f^ (46.92) *	17.29 ± 0.07 ^b^ (15.20) *
TAP	0	98.64 ± 12.23 ^b^	98.46 ± 5.69 ^a^	99.14 ± 2.30 ^c^	101.89 ± 3.50 ^a^
	1	112.67 ± 2.93 ^a^ (−14.22)	72.35 ± 4.16 ^b^ (26.52)	121.14 ± 3.60 ^ab^ (−22.19)	93.12 ± 9.72 ^ab^ (8.61)
	2	101.25 ± 0.32 ^b^ (−2.65)	65.32 ± 0.29 ^bc^ (33.66)	124.10 ± 0.74 ^a^ (−25.18)	87.18 ± 4.16 ^bc^ (14.44)
	3	107.32 ± 0.88 ^b^ (−8.80)	63.23 ± 0.46 ^c^ (35.78)	112.92 ± 3.02 ^b^ (−13.90)	79.28 ± 3.04 ^cd^ (22.19)
	4	100.55 ± 3.74 ^b^ (−1.94)	52.00 ± 6.87 ^d^ (47.19)	96.58 ± 5.27 ^c^ (2.58)	72.45 ± 0.46 ^de^ (28.89)
	5	92.03 ± 2.36 ^c^ (6.70) *	41.30 ± 2.38 ^e^ (58.05)	93.74 ± 1.77 ^c^ (5.45) *	61.35 ± 1.95 ^e^ (39.79) *
FF	0	293.80 ± 6.66 ^a^	203.04 ± 4.17 ^a^	199.29 ± 8.11 ^a^	101.83 ± 1.07 ^a^
	1	298.03 ± 9.48 ^a^ (−1.44)	165.75 ± 10.10 ^b^ (18.37)	159.73 ± 9.09 ^b^ (19.85)	83.15 ± 0.13 ^b^ (18.34)
	2	266.83 ± 9.51 ^b^ (9.18)	118.85 ± 0.26 ^c^ (41.46)	133.21 ± 3.41 ^c^ (33.16)	76.62 ± 0.21 ^b^ (24.76)
	3	251.20 ± 7.70 ^c^ (14.50)	115.11 ± 1.74 ^c^ (43.31)	113.79 ± 3.44 ^d^ (42.90)	75.91 ± 4.61 ^b^ (25.45)
	4	245.61 ± 3.32 ^c^ (16.40)	87.78 ± 6.67 ^d^ (56.77)	99.77 ± 1.73 ^e^ (49.94)	82.14 ± 9.93 ^b^ (19.34)
	5	208.57 ± 4.55 ^d^ (29.01) *	78.91 ± 5.40 ^d^ (61.14)	85.38 ± 3.14 ^e^ (57.16) *	80.40 ± 8.67 ^b^ (21.04) *
FFA	0	296.60 ± 9.87 ^a^	198.06 ± 6.02 ^a^	200.56 ± 3.89 ^a^	102.07 ± 3.53 ^a^
	1	285.17 ± 3.26 ^b^ (3.85)	198.81 ± 2.97 ^a^ (−0.38)	206.53 ± 5.50 ^a^ (−2.98)	82.21 ± 9.59 ^ab^ (19.46)
	2	239.38 ± 12.25 ^c^ (19.29)	160.87 ± 3.50 ^ab^ (18.78)	176.47 ± 6.06 ^ab^ (12.01)	60.34 ± 9.00 ^b^ (40.88)
	3	230.31 ± 12.66 ^cd^ (22.35)	146.54 ± 0.90 ^ab^ (26.01)	175.74 ± 16.52 ^ab^ (12.38)	79.34 ± 11.32 ^ab^ (22.27)
	4	220.12 ± 1.35 ^d^ (25.79)	121.13 ± 1.65 ^b^ (38.84)	168.28 ± 12.08 ^bc^ (16.09)	64.79 ± 8.90 ^b^ (36.52)
	5	174.13 ± 14.67 ^e^ (41.29) *	112.75 ± 2.89 ^b^ (43.07)	141.71 ± 6.47 ^c^ (29.34) *	60.97 ± 10.53 ^b^ (40.27) *

Different lowercase letters in the same column indicate significant differences (*p* < 0.05) in the concentration of the same compound in the same species of livestock and poultry meat between different deep-frying times. * indicates that the drug residue concentrations do not meet the requirements of non-detectable (CAP) or lower than MRLs (TAP, FF (sum of FF and FFA)), as stipulated by the Chinese standard GB 31650-2019 and (EU) No 37/2010 after deep-frying.

**Table 7:** Effect of microwave time on residues of amphenicols and metabolites in livestock and poultry meat.

Compound	Time (min)	Concentration (μg/kg) [Increase Rate (%)]			
		Pork	Beef	Lamb	Chicken
CAP	0	19.80 ± 1.66 ^d^	19.51 ± 1.04 ^c^	20.16 ± 0.63 ^a^	20.37 ± 0.64 ^b^
	0.25	20.73 ± 0.82 ^d^ (4.70)	21.39 ± 1.70 ^bc^ (9.64)	15.45 ± 1.25 ^b^ (−23.36)	18.25 ± 0.07 ^c^ (−10.41)
	0.50	23.07 ± 0.37 ^c^ (16.52)	22.53 ± 1.72 ^bc^ (15.48)	13.80 ± 0.28 ^b^ (−31.55)	18.05 ± 1.46 ^c^ (−11.39)
	0.75	26.29 ± 0.59 ^b^ (32.78)	27.73 ± 1.85 ^a^ (42.13)	15.29 ± 0.17 ^b^ (−24.16)	20.55 ± 1.18 ^b^ (0.88)
	1.00	26.57 ± 0.66 ^b^ (34.19)	24.86 ± 0.89 ^ab^ (27.42)	21.53 ± 0.93 ^a^ (6.80)	22.16 ± 0.71 ^b^ (8.79)
	1.25	28.48 ± 0.26 ^a^ (43.84) *	24.26 ± 0.87 ^abc^ (24.35) *	21.47 ± 2.77 ^a^ (6.50) *	25.80 ± 0.65 ^a^ (26.66) *
TAP	0	103.33 ± 7.46 ^b^	96.56 ± 0.93 ^b^	98.71 ± 3.20 ^d^	98.65 ± 2.66 ^d^
	0.25	99.33 ± 5.92 ^b^ (−3.87)	93.89 ± 6.13 ^b^ (−2.77)	128.82 ± 1.41 ^cd^ (30.50)	134.46 ± 4.19 ^c^ (36.30)
	0.50	102.42 ± 3.31 ^b^ (−0.88)	100.22 ± 12.31 ^b^ (3.79)	120.24 ± 2.39 ^cd^ (21.81)	145.52 ± 7.46 ^c^ (47.51)
	0.75	104.36 ± 5.91 ^ab^ (1.00)	123.88 ± 8.89 ^a^ (28.29)	141.51 ± 1.64 ^bc^ (43.36)	179.68 ± 9.72 ^b^ (82.14)
	1.00	105.41 ± 1.19 ^ab^ (2.01)	132.29 ± 14.44 ^a^ (37.00)	170.69 ± 7.32 ^ab^ (72.92)	179.58 ± 11.25 ^b^ (82.04)
	1.25	112.04 ± 2.90 ^a^ (8.43) *	128.69 ± 12.07 ^a^ (33.27) *	177.96 ± 2.97 ^a^ (80.29) *	232.74 ± 1.90 ^a^ (135.92) *
FF	0	286.87 ± 9.50 ^b^	198.55 ± 3.08 ^b^	200.68 ± 2.11 ^bc^	98.10 ± 1.76 ^d^
	0.25	303.27 ± 18.35 ^b^ (5.72)	192.76 ± 7.72 ^b^ (−2.92)	166.86 ± 14.41 ^cd^ (−16.85)	128.49 ± 12.96 ^c^ (30.98)
	0.50	305.81 ± 4.83 ^b^ (6.60)	190.03 ± 15.32 ^b^ (−4.29)	159.32 ± 2.47 ^d^ (−20.61)	145.82 ± 1.76 ^c^ (48.64)
	0.75	344.22 ± 5.64 ^a^ (19.99)	239.59 ± 18.25 ^a^ (20.67)	182.48 ± 12.85 ^cd^ (−9.07)	175.61 ± 6.35 ^b^ (79.01)
	1.00	357.21 ± 16.69 ^a^ (24.52)	264.34 ± 27.65 ^a^ (33.14)	227.29 ± 9.85 ^ab^ (13.26)	172.52 ± 11.96 ^b^ (75.86)
	1.25	334.82 ± 6.43 ^a^ (16.71) *	263.52 ± 17.13 ^a^ (32.72) *	237.40 ± 1.34 ^a^ (18.30) *	210.00 ± 12.32 ^a^ (114.07) *
FFA	0	300.40 ± 8.32 ^bc^	200.78 ± 4.87 ^c^	201.72 ± 1.51 ^b^	101.03 ± 1.22 ^d^
	0.25	290.38 ± 6.00 ^b^ (−3.34)	187.40 ± 13.56 ^cd^ (−6.66)	213.94 ± 2.68 ^b^ (6.06)	146.64 ± 17.16 ^c^ (45.15)
	0.50	309.49 ± 3.85 ^b^ (3.03)	175.94 ± 2.90 ^d^ (−12.37)	216.04 ± 0.03 ^b^ (7.10)	166.85 ± 27.39 ^bc^ (65.15)
	0.75	304.38 ± 4.52 ^bc^ (1.32)	203.23 ± 15.55 ^c^ (1.22)	270.97 ± 16.91 ^a^ (34.33)	213.37 ± 14.89 ^a^ (111.19)
	1.00	344.83 ± 12.91 ^a^ (14.79)	228.32 ± 0.54 ^b^ (13.72)	269.04 ± 0.85 ^a^ (33.37)	192.02 ± 15.40 ^ab^ (90.06)
	1.25	338.78 ± 6.50 ^a^ (12.78) *	247.29 ± 2.68 ^a^ (23.16) *	276.57 ± 6.09 ^a^ (37.11) *	179.19 ± 5.78 ^abc^ (77.36) *

Different lowercase letters in the same column indicate significant differences (*p* < 0.05) in the concentration of the same compound in the same species of livestock and poultry meat between different microwave times. * indicates that the drug residue concentrations do not meet the requirements of non-detectable (CAP) or lower than MRLs (TAP, FF (sum of FF and FFA)), as stipulated by the Chinese standard GB 31650-2019 and (EU) No 37/2010 after microwaving.

## Data Availability

Data are contained within the article.
